# Studies on Activities and Chemical Characterization of Medicinal Plants in Search for New Antimalarials: A Ten Year Review on Ethnopharmacology

**DOI:** 10.3389/fphar.2021.734263

**Published:** 2021-09-22

**Authors:** Isabela P. Ceravolo, Anna C. Aguiar, Joseph O. Adebayo, Antoniana U. Krettli

**Affiliations:** ^1^ Instituto René Rachou, Fundação Oswaldo Cruz (Fiocruz), Belo Horizonte, Brazil; ^2^ Departamento de Biociência, Universidade Federal de São Paulo, Santos, Brazil; ^3^ Department of Biochemistry, University of Ilorin, Ilorin, Nigeria

**Keywords:** malaria, antimalarials, medicinal plants, natural products, *in vivo* tests, ethnopharmacology

## Abstract

Malaria is an endemic disease that affected 229 million people and caused 409 thousand deaths, in 2019. Disease control is based on early diagnosis and specific treatment with antimalarial drugs since no effective vaccines are commercially available to prevent the disease. Drug chemotherapy has a strong historical link to the use of traditional plant infusions and other natural products in various cultures. The research based on such knowledge has yielded two drugs in medicine: the alkaloid quinine from Cinchona species, native in the Amazon highland rain forest in South America, and artemisinin from Artemisia annua, a species from the millenary Chinese medicine. The artemisinin-based combination therapies (ACTs), proven to be highly effective against malaria parasites, and considered as “the last bullet to fight drug-resistant malaria parasites,” have limited use now due to the emergence of multidrug resistance. In addition, the limited number of therapeutic options makes urgent the development of new antimalarial drugs. This review focuses on the antimalarial activities of 90 plant species obtained from a search using Pubmed database with keywords “antimalarials,” “plants” and “natural products.” We selected only papers published in the last 10 years (2011–2020), with a further analysis of those which were tested experimentally in malaria infected mice. Most plant species studied were from the African continent, followed by Asia and South America; their antimalarial activities were evaluated against asexual blood parasites, and only one species was evaluated for transmission blocking activity. Only a few compounds isolated from these plants were active and had their mechanisms of action delineated, thereby limiting the contribution of these medicinal plants as sources of novel antimalarial pharmacophores, which are highly necessary for the development of effective drugs. Nevertheless, the search for bioactive compounds remains as a promising strategy for the development of new antimalarials and the validation of traditional treatments against malaria. One species native in South America, *Ampelozyzyphus amazonicus*, and is largely used against human malaria in Brazil has a prophylactic effect, interfering with the viability of sporozoites in *in vitro* and *in vivo* experiments.

## Introduction

Human malaria is an infectious disease caused by single-cell protozoan parasites of the *Plasmodium* genus, namely: *P. falciparum*, *P. vivax*, *P. ovale*, *P. malariae*, and *P. knowlesi*, and is transmitted through the bite of female *Anopheles* mosquitoes (Crompton et al., 2014; Singh et al., 2021); of these, *P. falciparum* is the most virulent and prevalent globally. Symptoms can range from a mild to severe disease which can cause important physical disabilities and death, with an enormous health burden, especially among the most vulnerable and poor populations. While *P. falciparum* is responsible for most deaths, *P. vivax* is the most widespread of all of the Plasmodium species, can cause severe, even fatal infections and results in significant global morbidity and mortality (Menkin-Smith and Winders, 2020). Recent studies have reported the virulence of *P. vivax* (Lampah et al., 2011; Tanwar et al., 2011); although, the proportion of cases due to *P. vivax* reduced from about 7% of the global malaria cases in 2000 to 3% in 2019 (WHO, 2020a).

**FIGURE 1:**
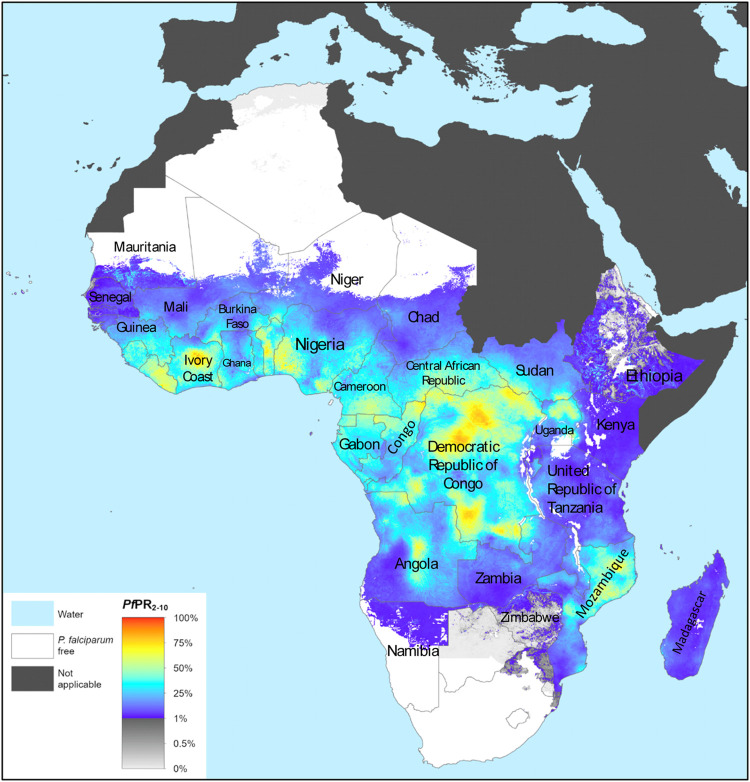
The spatial distribution of *Plasmodium falciparum* malaria endemicity in 2019 in the WHO African Region. Adapted from The Malaria Atlas Project (WHO, 2020b). Source: https://malariaatlas.org/trends/region.

**FIGURE 2:**
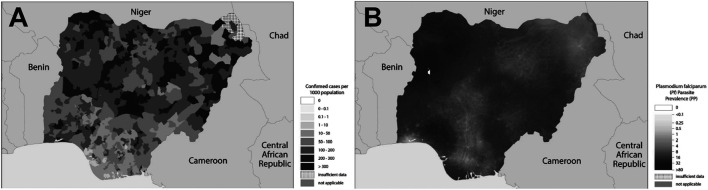
**(A)** Confirmed cases of malaria in Nigeria in 2017; **(B)** *Plasmodium falciparum* prevalence in Nigeria in 2017. Source: Adapted from WHO (2018a).

Malaria is an endemic disease in the tropical and subtropical regions of the world. An estimated 229 million new cases and 409,000 deaths were recorded in 2019 (WHO, 2020a). About 94% of the global malaria cases and 95% of the deaths were recorded in the African region in 2019 (Figure 1), with children under the age of 5 years and pregnant women being the most susceptible groups (WHO, 2020a). Nigeria was responsible for 27% of the malaria cases and 23% of deaths globally in 2019 (WHO, 2020a); this high prevalence illustrated in Figure 2 (WHO, 2018a). This is because Nigeria is the most populous nation in Africa, with *P. falciparum* being the main cause of malaria (Adebayo and Krettli, 2011; WHO, 2020a). Actually, *P. falciparum* is the most predominant species in every country in sub-Saharan Africa; the countries with the highest number of malaria cases in West, East, Central and Southern Africa are shown in Table 1. In some countries in Africa, a hundred percent of the population is at risk of the disease (both low and high), such as Nigeria, Mozambique, Democratic republic of Congo etc. (WHO, 2020a; Table 1). However, the numbers of reported cases in such countries are low compared to the expected numbers because of lack of access to diagnosis/health facilities or presumptuous treatment (Table 1). Ethiopia has been reported to have the highest number of *P. vivax* infections in Africa (WHO, 2020a). The higher prevalence of the disease based on the reported cases in the other regions of Africa compared to Southern Africa (Table 1) is an indication of favorable environmental conditions such as higher temperature and rainfall in such regions (Greenwood et al., 2008).

**TABLE 1:** Population, estimated malaria cases and deaths in selected countries with the highest malaria cases in different African regions in 2019.

Region of Africa	Country	Total population	Population at risk (% of total population)	Cases (% of population at risk)	Deaths	Most predominant *Plasmodium* species
West Africa	Nigeria	200,963,608	200,963,608 (100)	60,959,012 (30.3)	95,418	*Plasmodium falciparum*
East Africa	Mozambique	30,366,043	30,366,043 (100)	9,364,806 (30.8)	14,971	*P. falciparum*
	Ethiopia	112,078,736	76,213,540 (68)	2,614,852 (3.4)	5,626	*P. falciparum*, coupled with the highest number of *P. vivax* cases in Africa
Central Africa	Democratic Republic of Congo	86,790,564	86,790,564 (100)	28,280,007 (32.6)	44	*P. falciparum*
Southern Africa	Namibia	2,494,524	1,980,028 (79.4)	5,618 (0.3)	14	*P. falciparum*

Source: Adapted from WHO (2020a).

Drug chemotherapy has a strong historical link to the use of traditional plant infusions in various cultures. Research based on such knowledge has yielded the two most important drugs critical to malaria: the alkaloid quinine present in *Cinchona* species of highland rain forests in South America and artemisinin from *Artemisia annua*, first registered in the millenary Chinese medicine.

Artemisinin-based combination therapies (ACTs) are the most recommended treatments for uncomplicated *P. falciparum* malaria, while artesunate is considered the most effective antimalarial drug for severe cases (Roussel et al., 2017), with several biochemical processes reported as targets in the parasites (Wicht et al., 2020). Despite the safety and efficacies proven for the use of these antimalarial drugs, the emergence of multidrug resistance has unfortunately limited their effects and challenged the field (Nieves et al., 2016). The resistance to ACTs is already spreading from Southeast Asia as reported in 2008 (Noedl et al., 2008), giving rise to a danger alert to other high-poverty regions in the world. The identified artemisinin resistance phenotype is associated with mutation of a kelch domain protein gene (k13), postulated to be involved in the parasite protein trafficking organelles during the intraerythrocytic cycle (Ariey et al., 2014). This mutation has recently been reported in East Africa (Uwimana et al., 2020; Asua et al., 2021), Papua New Guinea (Miotto et al., 2020), and French Guiana (Mathieu et al., 2020); yet, there is limited evidence of delayed parasite clearance after ACT treatment in these regions (Yeka et al., 2016).

The treatment failure associated with ACTs in Africa has been linked to resistance of parasite to the partner drugs and not to artemisinin resistance (Slater et al., 2016). However, Ala675Val mutation in the kelch 13 propeller gene was seen in 5% of isolates in Northern Uganda and this mutation has been known to cause delayed parasite clearance in Southeast Asia (Asua et al., 2019). In the African continent, four countries have identified mutations associated with therapeutic failure of artemisinin derivatives. However, *Pfk13* mutations (M476I, P553L, R561H, P574L, C580Y, and A675V) showed low frequencies and with no reports of clinical treatment failure, except in Rwanda (Miotto et al., 2020; Uwimana et al., 2020; Asua et al., 2021).

Due to the resistance and the high cost of the potent ACTs, the poor populations of the African continent rely heavily on herbal remedies based on historical and cultural beliefs (Adebayo and Krettli, 2011). In addition, the limited number of therapeutic options makes the development of new antimalarial drugs urgent. The search for bioactive compounds from plants remains a promising strategy for the development of new antimalarial candidates as well as the validation of traditional treatments. In such context, the aim of this study was to review the literature for activities and safety of antimalarial plants *in vivo* and their isolated active principles studied during the last 10 years.

### History of Natural Products and Their Applications in the Treatment of Human Malaria

The history of medicine dates back to the existence of human civilization. Most new drugs have been generated from natural products (secondary metabolites) and from compounds derived from natural products (Patwardhan et al., 2004; Lahlou, 2007). Natural products from plants, animals, and minerals, have been the basis of treatment of human diseases (Lahlou, 2007); nevertheless, plants used in traditional medicine may hold the key of many potent antimalarial drugs (Rasoanaivo et al., 2004).

Studies of plants used in traditional medicine for the treatment of malaria in various cultures have yielded important findings that are crucial to modern medicine. Two of the most effective drugs for malaria originated from traditional medicine: quinine from bark of Peruvian *Cinchona* tree, and artemisinin from the Chinese antipyretic *Artemisia annua* (Rasoanaivo et al., 2004).

Currently, standardized antimalarial phytomedicines are officially commercialized in various malaria endemic countries over the world, namely China, Ghana, India, Mali, and Burkina Faso (Abay et al., 2015), supporting their use as complementary tools to the conventional antimalarial interventions or as alternative treatments in the absence of antimalarial drugs (Willcox, 2011). Examples of herbal therapies are represented by Qing hao (*Artemisia annua*, Democratic Republic of Congo trials), Totaquina (*Cinchona* spp., Multicounty trials) and Phyto-laria (*Cryptolepis sanguinolenta*, Ghana trial) (Willcox, 2011). Medicinal plants are also used to treat malaria in several African countries (Soh and Benoit-Vical, 2007; Pillay et al., 2008; Adebayo and Krettli, 2011; Maranz, 2012; Chinsembu, 2015; Memvanga et al., 2015; Omara et al., 2020). In Ethiopia, 80% of the rural populations rely on medicinal plants to treat diseases due to the high cost of drugs, low access to health services, among other factors. As a result, many species of medicinal plants are used against malaria there, such as *Artemisia annua*, *Ajuga remota*, *Azadirachta indica*, *Argemone mexicana*, *Vernonia amygdalina*, *Asparagus africanus*, *Uvaria leptocladon*, and *Gossypium* spp (Alebie et al., 2017).

### Quinine

Historical records show that in 1638, the countess of Chinchón, wife of the Spanish viceroy in Peru, suffered a severe fever, which was later known as malaria. When ingesting a portion made by the Indians called “quina-quina” the fever subsided, and the continuity of treatment left her cured (Willcox et al., 2004; Ruiz-Irastorza and Khamashta, 2008; de Oliveira and Szczerbowski, 2009). There is also a reference that the use of *Cinchona* species in medical practice came to Belgium in 1643, when a public health official in Ghent recommended a powder for the treatment of tertian fevers (Jarcho and Torti, 1993). However, the first indisputable reference is the Schedula Romana, a hand bill issued by the Pharmacy of the Collegio Romano in 1649 and again in 1651, containing precise instructions on its dosage and administration (Jaramillo-Arango, 1949).

Before 1820, the bark of a tree native to South America named *Cinchona*, was dried, ground to a fine powder, and mixed into a liquid (commonly wine) before being used (Achan et al., 2011). The Jesuit priests of the Spanish mission took the tree bark to Europe to sell its powder as medicine against intermittent fevers. Later on, two French chemists, Pelletier and Caventou (1820), isolated the active principle by precipitation and crystallization and discovered that the base febrifuge compounds were the alkaloids named cinchonine and quinine. From it, the alkaloids quinidine and cinchonidine were also described (Willcox et al., 2004; Kaufman and Ruveda, 2005). The four alkaloids from *Cinchona* bark were all effective against malaria, but quinidine and quinine possessed equal febrifugal activity, while cinchonidine was slightly less efficacious. An additional 25 alkaloids related to quinine had been isolated by 1884 and six more were isolated between 1884 and 1941 (Turner and Woodward, 1998). By the turn of the 18th and 19th centuries, *Cinchona* bark and quinine became widely accepted and used instead of the powdered bark as the reference treatment for intermittent fevers throughout the world (Gachelin et al., 2017).

**FIGURE 3:**
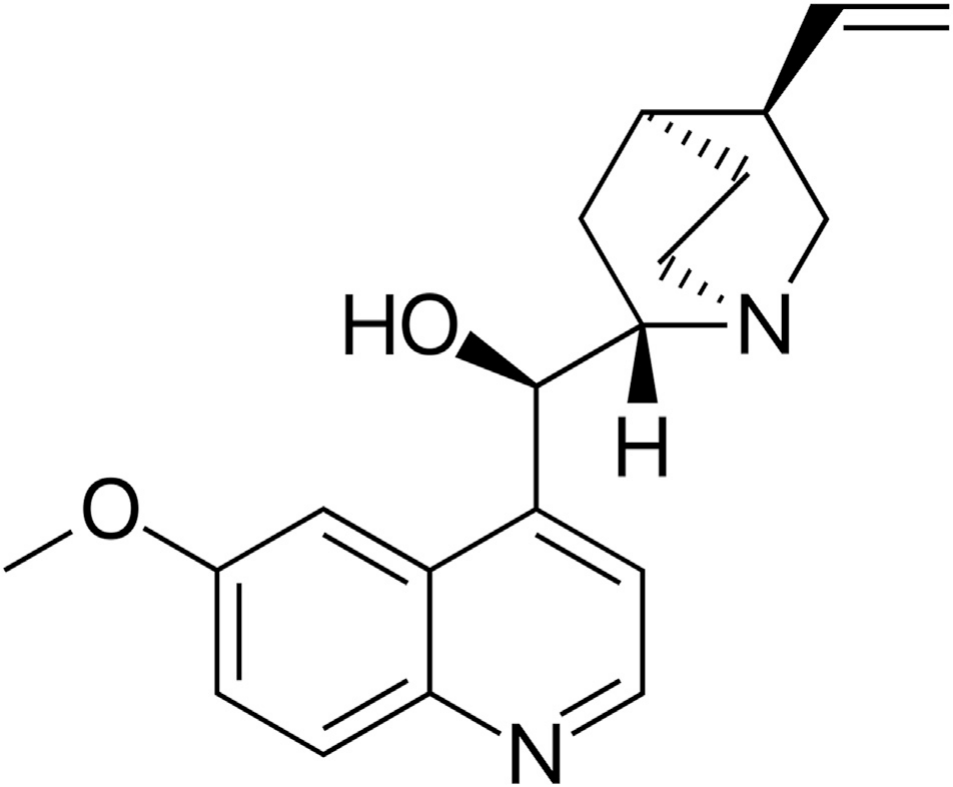
Chemical structure of quinine. Source: https://commons.wikimedia.org/wiki/File:Quinine_structure.svg.

During the second World War, there was an interruption in the supply of *Cinchona* bark, which contained a high amount of quinine, from the Java island where the seeds were largely cultivated (Howard, 1931). In the mid-1800s, the structure of quinine (Figure 3) was elucidated (Kaufman and Ruveda, 2005). Quinine remained the mainstay of malaria treatment until the 1920s, when more effective synthetic antimalarial drugs became available (AdenAbdi et al., 1995). However, the extraction of quinine from the bark of *Cinchona* does not yield as much as the extraction from the entire tree. The commercial achievement of this bark, almost led to the extinction of the Amazonian trees, and therefore, there was a great need to synthesize the active compounds (Smith and Willliams, 2008).

Quinine is an alkaloid that presents rapid schizonticidal action against intra-erythrocytic malaria parasites; it is also gametocytocidal for *Plasmodium vivax* and *Plasmodium malariae*, though there are conflicting reports on its gametocytocidal effect on *Plasmodium falciparum* (Chutmongkonkul et al., 1992; Tanaka and Williamson, 2011). Quinine has also an analgesic effect, but not antipyretic properties, and prevents the hemozoin crystals from growing by intercalating the quinolone rings between the aromatic groups of the ferriprotoporphyrine molecules (Pelletier and Caventou, 1820; Institute of Medicine, 2004; Weissbuch and Leiserowitz, 2008; Gachelin et al., 2017). For centuries, quinine has been used to treat malaria, and it remains the drug of choice in the treatment of severe malaria; it has been chosen as a second line treatment in combination with antibiotics, after artemisinin combination therapy (ACT) in the treatment of complicated malaria (WHO, 2015).

### Resistance to Quinine

Parasite drug resistance is probably the greatest problem faced by malaria control programs worldwide and is an important public health concern. Over the years, malaria parasites have developed resistance to several commonly used antimalarial drugs (Achan et al., 2011). Diminished sensitivity of *P. falciparum* to quinine has been widely documented in Asia (Sucharit et al., 1979; Bjorkman and Phillips–Howard, 1990; Mayxay et al., 2007), South America (Neiva, 1987; Best Plummer and Pinto Pereira, 2008; Legrand et al., 2008), and Africa (Pradines et al., 1998; Zalis et al., 1998; Mutanda, 1999 *).* However, the development of resistance to quinine has been slow; although its use started in the 17th century, resistance to quinine was first reported in 1910 (Peters, 1982), and after so many years of its discovery, quinine remains an important and effective treatment for malaria today, despite sporadic reports of resistance (Institute of Medicine, 2004). Thus, with increasing resistance in almost all areas with *P. falciparum* malaria to synthetic antimalarials by the 1980s, quinine again played a key role, particularly in the treatment of severe malaria (AdenAbdi et al., 1995).

Quinine is still used as monotherapy to treat malaria today in most countries of Africa and in some Central American, Caribbean, Eastern Mediterranean, South-East Asian and Western Pacific countries (WHO, 2019). In several settings, the use of quinine for uncomplicated malaria cases has reduced due to toxicity, poor compliance, and the implementation of newer and better tolerated therapies. However, from frequent stock-outs of the recommended ACT and the increasing resistance to chloroquine and antifolates, quinine use in recent times has increased (Wasunna et al., 2008; UMSP, 2010).

The 2015 World Health Organization (WHO) guidelines recommend a combination of quinine and clindamycin, or only quinine to treat pregnant women with uncomplicated *P. falciparum* and who have chloroquine-resistant *P. vivax* malaria during the first trimester. For the treatment of severe malaria of adults and children, it is essential that full doses of effective parenteral (or rectal) antimalarial treatment be given promptly in the initial treatment (WHO, 2015). The *Cinchona* alkaloids quinine and quinidine are the second choice, after artemisinin derivatives. When a complete oral treatment of severe malaria is not possible, but injections are available, the intramuscular quinine could be used if artesunate or artemether is not available (WHO, 2015).

### Artemisinin

Qinghao (“blue-green herb”) is the Chinese name for a relatively common herbal plant otherwise known as *Artemisia annua* or sweet wormwood (Maude et al., 2010). *Artemisia annua* is native to Asia (China, Japan, Korea, Vietnam, Myanmar, Northern India, Southern Siberia and throughout eastern Europe) (WHO, 1998), but it also grows in other countries such as Congo, India, Brazil, Australia, Argentina, Bulgaria, France, Hungary, Italy, Spain, and the USA (Willcox et al., 2003; Zhou et al., 2014). There is an integral interaction between plants, the environment and the production of secondary metabolites, which can directly interfere with their quantities and qualities (Kutchan, 2001). The artemisinin content is affected by several factors, such as geographic conditions, harvest time, temperature and fertilizer application; harvesting the plant at the right time is extremely important to ensure the optimal artemisinin content in *A. annua*. Thus, for the cultivation of this medicinal plant, it is important to plan the crop establishment for the beginning of the rainy season, and the soil needs to be plowed to a fine slope and consolidated by rolling when appropriate, because the sowing depth is also critical for *A. annua* (Jelodar et al., 2014). This means that not all *A. annua* plants have high concentrations of antimalarial compounds.

**FIGURE 4:**
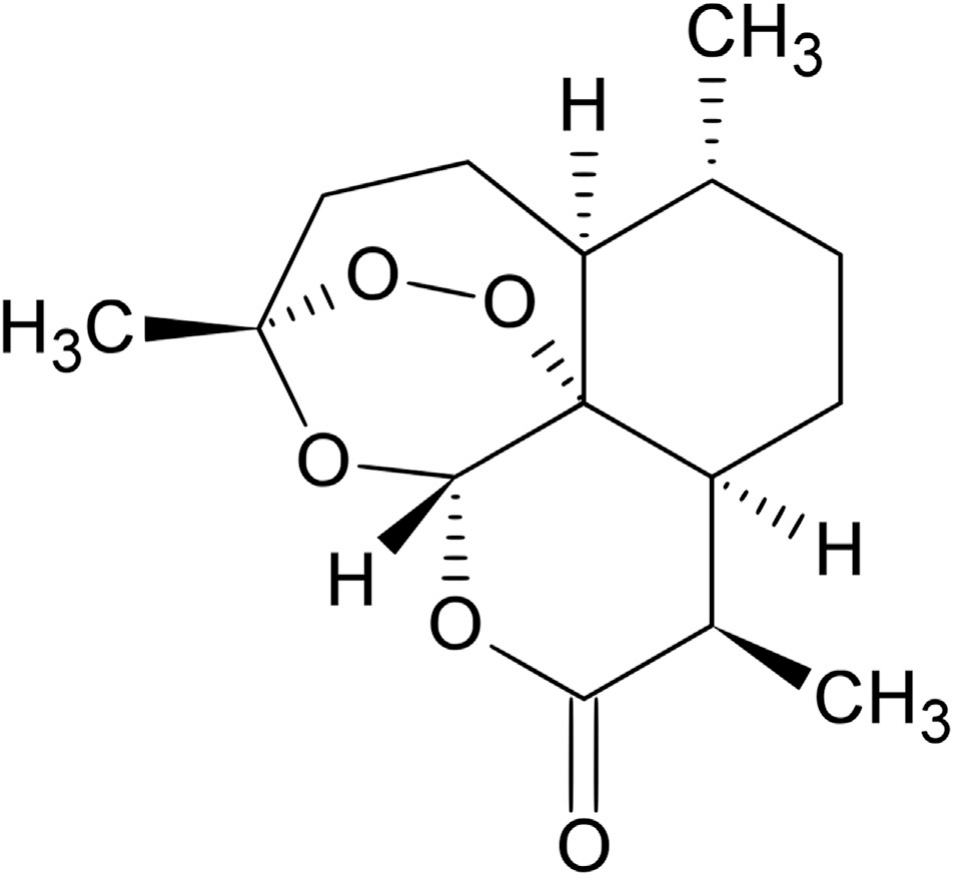
Chemical structure of artemisinin. Source: https://en.wikipedia.org/wiki/Artemisinin.

The *A. annua* has been used as a remedy by Chinese herbalists for the crafting of aromatic wreaths, as a source of essential oils used in flavoring vermouth, and for more than 2,000 years in Chinese traditional medicine, as a treatment for fever and hemorrhoids (Van Agtmael et al., 1999; Klayman, 1985). *Artemisia annua* is also used as a source of artemisinin (Zhou et al., 2014), a potent antimalarial drug discovered by the Chinese scientists led by Youyou Tu, that was awarded the 2011 Lasker Prize and the Nobel Prize in Physiology or Medicine in 2015 (Youyou, 2015). The project leading to the discovery of artemisinin was initiated in response to a request from North Vietnamese leaders who were suffering heavy losses of soldiers due to malaria during the Vietnam War (Cui and Su, 2009). The drug is present in the leaves and flowers of the plant accounting for approximately 0.01–0.8% of dry weight (Van Agtmael et al., 1999). Chemically, artemisinin is a sesquiterpene trioxane lactone containing a peroxide bridge (Figure 4), which is essential for its activity (China Cooperative Research Group, 1982).

The earliest known record of *A. annua* was in the book *52 Prescriptions*, discovered in the Mawangdui tomb of the Han Dynasty in 168 BC, where it was first described for the treatment of hemorrhoids (Qinghaosu Antimalarial Coordinating Research Group, 1979; Klayman, 1985). Around 340 AD, in Hong Ge’s Handbook of Prescriptions for Emergency Treatment, a cold extraction method of qinghao was described for the treatment of intermittent fevers (Klayman, 1985). Qinghao has also been mentioned in several later standard Chinese Materia Medica texts as treatment for fever (Qinghaosu Antimalaria Coordinating Research Group, 1979; Hien and White, 1993); it has also been used for haemorrhoids, lice, wounds, boils, sores, and convulsions (Hsu, 2006).

The active ingredient of qinghao, artemisinin, was isolated from the plant *A. annua* in 1972 by a group of Chinese scientists (Hien and White, 1993). In 1975, its unique chemical structure was elucidated, as a sesquiterpene lactone bearing a peroxy group, which is believed to be responsible for the antimalarial activity of *A. annua* (Willcox et al., 2003), and is quite different from those of all known antimalarial drugs (Liu et al., 1979), then the name qinghaosu (“active principle of qinghao”) was changed to artemisinin (Klayman, 1985). Its structure was determined by X-ray analysis in 1979 (Balint, 2001; Krishna et al., 2008). Further studies led to the development of more stable derivatives, such as dihydroartemisinin, artemether, artemotil (arteether) and artesunate (Ansari et al., 2013).

Artemisinin and its derivatives produce rapid parasite clearance, killing young circulating parasites more than the other forms of the parasite, before they are sequestered in the deep microvasculature (Dondorp and Von Seidlein, 2017). This phenomenon is important in the pathogenesis of malaria because mature parasites are able to adhere to endothelial cells, blood cells and platelets, which prevent their circulation in the bloodstream and they will therefore be able to escape clearance by the spleen (Buffet et al., 2011). This is clinically relevant, particularly in the cases of cerebral and severe malaria (Hsu, 2006). These drugs interact with heme to produce carbon-centered free radicals that alkylate protein and damage the microorganelles and membranes of the parasites (Meshnick, 1998), significantly faster than any other antimalarial does (Willcox et al., 2003; Sriram et al., 2004). Unlike the quinine-related drugs and antifolate drugs, artemisinin and its derivatives are also gametocytocidal and reduce the transmission of malaria by *P. falciparum* (Price et al., 1996).

Since 1994, artemisinins have been used in artemisinin-based combination therapies (ACTs) to treat uncomplicated malaria (Ouji et al., 2018), and by 2006, ACTs had become the recommended treatments for falciparum malaria worldwide (WHO, 2015). The artemisinin derivatives are usually combined with a more slowly eliminated drug in ACTs (Ansari et al., 2013). In this case, the role of the artemisinin compound is to reduce the number of parasites during the first 3 days of treatment (reduction of parasite biomass), while the role of the partner drug is to eliminate the remaining parasites leading to a malaria cure. ACTs are also recommended by WHO for chloroquine-resistant *P. vivax* malaria, and the injectable artesunate and artemether, are recommended for the treatment of severe malaria (WHO, 2018b).

ACTs are the most effective antimalarial medicines available today, but there are already cases of resistance to artemisinin and its derivatives. Artemisinin resistance in Cambodia was first identified in clinical studies in 2006, and few years later in Myanmar, Thailand, Viet Nam, and Lao. However, retrospective analysis of molecular markers indicated that artemisinin resistance probably emerged in 2001, before the widespread deployment of ACTs in Cambodia (Noedl et al., 2008; Dondorp et al., 2009; WHO, 2017). In spite of the fact that WHO continues to monitor cases of resistance to artemisinin and its derivatives, new drugs must be discovered to overcome the problem of the development of resistance that can make its use unfeasible in the future.

### Current Malaria Treatment

ACTs are recommended by WHO as the first-and second-line treatment for uncomplicated *P. falciparum* malaria, as well as for chloroquine-resistant *P. vivax* malaria. The role of the artemisinin compound is to reduce the number of parasites during the first 3 days of treatment (reduction of parasite biomass), while the role of the partner drug is to eliminate the remaining parasites, leading to cure (WHO, 2018b). It is recommended to use a 3-day treatment regimen with one of the five different ACTs, namely artemether/lumefantrine, artesunate/amodiaquine, artesunate/mefloquine, dihydroartemisinin/piperaquine or artesunate/sulfadoxine-pyrimethamine. ACTs are not recommended in the first trimester of pregnancy for uncomplicated *P. falciparum* malaria; the World Health Organization guidelines (WHO, 2015) recommends a combination of quinine and clindamycin, or only quinine for uncomplicated malaria caused by *P. falciparum* and by chloroquine-resistant *P. vivax*.

In areas with chloroquine-sensitive *P. vivax*, *P. ovale*, *P. malariae* or *P. knowlesi* infections, the adults and children are treated with ACTs or chloroquine. Primaquine can be administered to avoid relapses of vivax malaria in people with no Glucose-6-phosphate dehydrogenase deficiency. The treatment of adults and children with severe malaria is done with intravenous or intramuscular artesunate for at least 24 h, but if parenteral artesunate is not available, intramuscular artemether is used in preference to quinine (WHO, 2015).

## Methods

### Study Selection and Analysis

**FIGURE 5:**
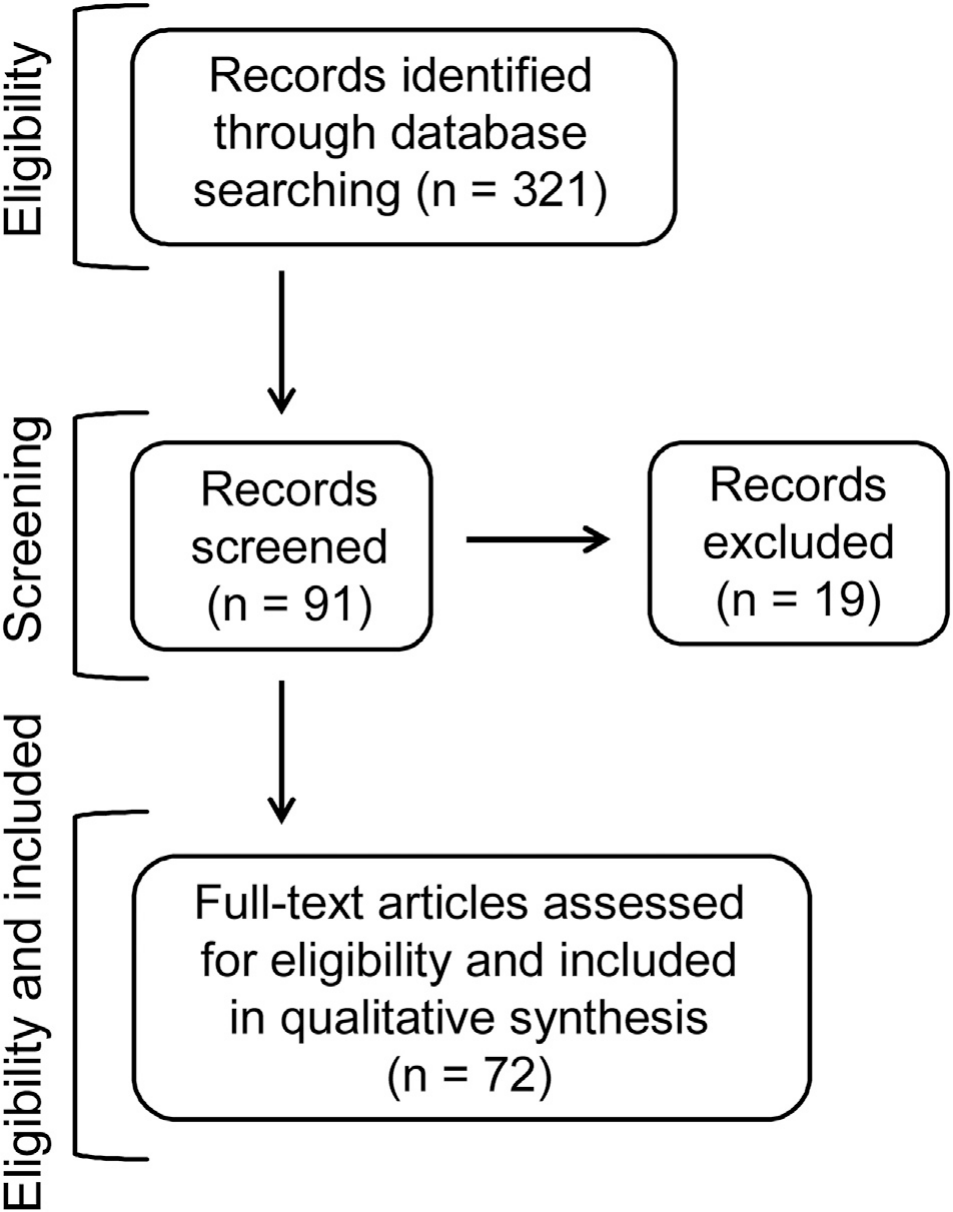
Flowchart of literature search and selection criteria used in the present review.

The flowchart (Figure 5) demonstrates the strategy used for identification, and the criteria for inclusion and exclusion of the plants focused in the present review. A total of 321 articles were initially retrieved from the database Pubmed in the last 10 years (2011–2020) using the keywords “antimalarials,” “plants” and “natural products.” Some additional references of other articles published before have been also included, although they were not found after using the keywords. In a further analysis, articles which focused on the plant species tested *in vivo* against malaria, using mice infected with *Plasmodium* species, were selected. This gave a total of 91 full articles, from which 19 articles were excluded because: 1) they were in other languages rather than English; 2) the reported chemosuppression was below 30%, which did not correspond to the criteria for antimalarial activity, as defined below; 3) the tests were with polyherbal extracts rather than with one species of plant; 4) the active plants were toxic; 5) the tests against early sporogonic stages were conducted *ex vivo*; or, 6) the studies were performed using plants associated or in combination with antimalarial drugs. After applying all these requirements, the total number of publications evaluated was 72, as shown in Table 2 and Figure 5.

**TABLE 2:** Botanical classification, ethnomedical uses of plants evaluated for antimalarial activity in mice infected with *Plasmodium* species, and countries of studies from 2011 to 2020.

Family^a^	Plant species	Part(s) used	Ethnomedical use	Country	Reference
Acanthaceae (2)	*Acanthus polystachyus*	Roots, leaves	Malaria and others	Ethiopia	Derebe and Wubetu (2019), Kifle and Atnafie (2020)
	*Hypoestes forskalei*	Leaves	Malaria, antipyretic, antileishmanial, antitrypanosomal	Ethiopia	Misganaw et al. (2020)
Amaranthaceae (1)	*Chenopodium ambrosioides*	Leaves	Anti-inflammatory, anti-*Leishmania* and others	Brazil	Cysne et al. (2016)
Anacardiaceae (2)	*Adansonia digitata*	Stem bark	Malaria	Kenya	Musila et al. (2013)
	*Sorindeia juglandifolia*	Fruits	Not found	Cameroon	Kamkumo et al. (2012)
Annonaceae (2)	*Polyalthia longifólia*	Leaves	Malaria	Nigeria	Bankole et al. (2016)
	*Xylopia amazonica*	Leaves, branches	Not found	Brazil	Lima et al. (2015)
Apiaceae (9)	*Daucus virgatus*	Aerial parts	Not found	Tunisia	Sirignano et al. (2019) ^b^
	*Ferulago angulata*	Aerial parts	Sedative, tonic, and parasitic effects	Iran	Sajjadi et al. (2016)
	*Aspidosperma nitidum*	Wood bark, leaves, branches	Malaria	Brazil	Coutinho et al. (2013)
	*Aspidosperma olivaceum*	Stem bark, leaves	Fevers	Brazil	Chierrito et al. (2014)
	*Aspidosperma pyrifolium*	Stem bark, stem	Inflammation process and dermatitis	Brazil	Ceravolo et al. (2018)
	*Aspidosperma ramiflorum*	stem bark, leaves	Not found	Brazil	Aguiar et al. (2015)
	*Calotropis gigantea*	Leaves, stems, flowers	Several non-antimalarial effects	Ethiopia	Satish et al. (2017)
	*Holarrhena pubescens*	Roots	Malaria	Tanzania	Nondo et al. (2016)
	*Periploca linearifolia*	Stem bark	Malaria	Ethiopia	Belay et al. (2018)
Apocynaceae (1)	*Acokanthera schimperi*	Leaves	Malaria	Ethiopia	Mohammed et al. (2014)
Arecaceae (1)	*Euterpe oleracea*	Fruit pulp	Anti-inflammatory and others effects	Brazil	Ferreira et al. (2019)
Asphodelaceae (4)	*Aloe sp*	Leaves	Malaria	Ethiopia	Mesfin et al. (2012)
	*Aloe pirottae*	Leaves	Malaria	Ethiopia	Dibessa et al. (2020)
	*Aloe weloensis*	Leaf latex	Malaria and several others diseases	Ethiopia	Teka et al. (2020)
	*Kniphofia foliosa*	Rhizomes	Abdominal cramps and wound healing	Ethiopia	Induli et al. (2013)
Asteraceae (4)	*Dicoma tomentosa*	Whole plant	Malaria	Burkina Faso	Jansen et al. (2012)
	*Echinops hoehnelii*	Roots	Malaria	Ethiopia	Bitew et al. (2017)
	*Echinops Kebericho*	Rizomes, roots	Malaria, fevers and others	Ethiopia	Toma et al. (2015), Biruksew et al. (2018)
	*Helianthus annuus*	Roots, stems, seeds, flowers, leaves	Malaria and others diseases	Indonesia	Ekasari et al. (2019), Ekasari et al. (2020)
Bignoniaceae (1)	*Markhamia tomentosa*	Fresh leaves	Malaria	Nigeria	Bankole et al. (2016)
Bombacaceae (1)	*Bombax buonopozense*	Root bark	Malaria, pain, fevers and diarrhoea	Nigeria	Christian et al. (2017)
Boraginaceae (1)	*Cordia africana*	Leaves	Malaria	India	Wondafrash et al. (2019)
Combretaceae (2)	*Terminalia brownii*	Barks	Malaria	Ethiopia	Biruk et al. (2020)
	*Terminalia macroptera*	Leaves, roots	Malaria and others diseases	Mali	Haidara et al. (2018)
Cucurbitaceae (3)	*Citrullus colocynthis*	Fruits	Malaria	Iran	Haddad et al. (2017)
	*Cucumis metuliferus*	Leaves	Malaria	Tanzania	Mzena et al. (2018)
	*Gynostemma pentaphyllum*	Leaves	Several diseases other than malaria	Thailand	Somsak et al. (2016)
Euphorbiaceae (2)	*Croton macrostachyus*	Leaves	Malaria	Ethiopia	Bantie et al. (2014), Mohammed et al. (2014)
	*Phyllanthus nivosus*	Leaves	Malaria, fevers, headaches, toothaches, tooth infections	Nigeria	Johnson et al. (2020)
Fabaceae (11)	*Acacia karroo*	Leaves	Pyretic diseases	India	Sachdeva et al. (2020)
	*Acacia nilotica*	Roots	Malaria	Nigeria	Alli et al. (2016)
	*Caesalpinia bonducella*	Roots	Malaria	Tanzania	Nondo et al. (2016)
	*Caesalpinia pluviosa*	Stem bark	Antiviral and other infections	Brazil	Kayano et al. (2011)
	*Commiphora africana*	Stem bark	Malaria and other diseases	Tanzania	Kweyamba et al. (2019)
	*Copaifera reticulata*	Oleoresin	Anti-inflammatory and other properties	Brazil	de Souza et al. (2017)
	*Dichrostachys cinérea*	Stem bark, whole stem	Malaria and other diseases	Tanzania	Kweyamba et al. (2019)
	*Glycyrrhiza glabra*	Roots	To reduce the toxicity and enhances effectiveness of other drugs	India	Kalani et al. (2013)
	*Indigofera spicata*	Roots	Malaria	Ethiopia	Birru et al. (2017)
	*Pongamia pinnata*	Leaves, bark, flower, root	Antiplasmodial, antioxidant and other effects	India	Satish and Sunita (2017)
	*Tamarindus indica*	Fruits	Malaria	Ethiopia	Mesfin et al. (2012)
Gentianaceae (1)	*Anthocleista djalonensis*	Stem bark	Not found	Ivory Coast	Attemene et al. (2018)
Icacinaceae (1)	*Icacina senegalensis*	Root bark	Antimalaria, antimicrobial and others	Nigeria	Akuodor et al. (2017)
Lamiaceae (7)	*Ajuga bracteosa*	Leaves	Malaria and other effects	India	Chandel and Bagai (2011)
	*Ajuga integrifólia*	Aerial parts	Malaria	Ethiopia	Asnake et al. (2015)
	*Leonotis ocymifolia*	Leaves	Malaria, yellow fever and others	Ethiopia	Teklu et al. (2020)
	*Ocimum lamifolium*	Leaves	Malaria	Ethiopia	Kefe et al. (2016)
	*Ocimum sanctum*	Leaves	To enhance immunity and others effects	India	Rajendran et al., 2014
	*Ocimum suave*	Leaves	Malaria	Kenya	Kiraithe et al. (2016)
	*Plectranthus barbatus*	Root bark	Malaria	Kenya	Kiraithe et al. (2016)
Meliaceae (4)	Azadirachta indica	Leaves	Malaria	Ethiopia	Mesfin et al. (2012)
	*Entandrophragma cylindricum*	Stem bark	Malaria, yellow fever and other effects	Cameroon	Nadia et al. (2020)
	*Melia azedarach*	Twigs	Malaria	Ethiopia	Asnake et al. (2015)
	*Trichilia heudelotii*	Stem bark	Malaria	Nigeria	Bankole et al. (2016)
Moraceae (1)	*Ficus thonningii*	Leaves	Several uses, non-antimalarial	Nigeria	Falade et al. (2014)
Moringaceae (1)	*Moringa oleifera*	Leaves	Antioxidant and other effects	Thailand	Somsak et al. (2016)
Myricaceae (1)	*Myrica salicifolia*	Roots	Malaria	Ethiopia	Kifle et al. (2020)
Myrtaceae (2)	*Psidium guajava*	Leaves, unripe fruits	Broad spectrum of activities	India	Rajendran et al. (2014)
	*Syzygium cumini*	Leaves	Pyretic diseases	India	Sachdeva et al. (2020)
Ochnaceae (1)	*Lophira alata*	Leaves	Anti-fever and other uses	Nigeria	Falade et al. (2014)
Oleaceae (1)	*Olea europaea*	Stem bark, leaves	Malaria and other infections	Ethiopia	Misganaw et al. (2019), Hailesilase et al. (2020)
Picramniaceae (1)	*Picramnia latifolia*	Bark/petiole	Not found	Colombia	Berthi et al. (2018)
Piperaceae (1)	*Piper peltatum*	Roots	Malaria	Brazil	Rocha e Silva et al. (2011)
Poaceae (1)	*Andropogon leucostachyus*	Aerial parts	Malaria	Brazil	Lima et al. (2015)
Ranunculaceae (1)	*Coptis japônica*	Rhizome	Inflammatory disease	Japan	Teklemichael et al. (2020)
Rhamnaceae (1)	*Ziziphus mauritiana*	Leaves	Not found	Ivory Coast	Attemene et al. (2018)
Rosaceae (1)	*Rubus ellipticus*	Leaves	Pyretic diseases	India	Sachdeva et al. (2020)
Rubiaceae (3)	*Canthium glaucum*	Roots	Malaria	Kenya	Musila et al. (2013)
	*Gardenia ternifolia*	Root bark	Malaria	Ethiopia	Nureye et al. (2018)
	*Heinsia crinita*	Leaves, fruits, stem barks	Malaria, fever and others	Congo	Tshisekedi et al. (2017)
Rutaceae (3)	*Fagara zanthoxyloides*	Leaves	Malaria	Nigeria	Enechi et al. (2019)
	*Murraya koenigii*	Leaves	Malaria and to decrease insulin level	India	Kamaraj et al. (2014), Rajendran et al. (2014)
	*Zanthoxylum chalybeum*	Root bark	Malaria	Kenya	Kiraithe et al. (2016)
Sapindaceae (1)	*Dodonaea angustifólia*	Roots	Malaria	Ethiopia	Amelo et al. (2014)
Santalaceae (1)	*Osyris quadripartita*	Leaves	Malaria	Ethiopia	Girma et al. (2015)
Simaroubaceae(1)	*Picrolemma huberi*	Cortex	Not found	Colombia	Berthi et al. (2018)
Solanaceae (2)	*Physalis alkekengi*	Leaves and fruits	Anti-fever and other uses	Iran	Haddad et al. (2017)
	*Solanum nigrum*	Fruits	Not found	Iran	Haddad et al. (2017)
Stilbaceae (1)	*Nuxia congesta*	Leaves	Malaria	Ethiopia	Fenta and Kahaliw (2019)
Strychnaceae (1)	*Strychnos mitis*	Leaves	Malaria	Ethiopia	Fentahun et al. (2017)
Tiliaceae (1)	*Grewia trichocarpa*	Roots	Malaria	Kenya	Mwangi et al. (2015)
Verbenaceae (1)	*Lippia kituiensis*	Leaves	Malaria	Tanzania	Mzena et al. (2018)
Zingiberaceae (1)	*Zingiber officinale*	Roots	Malaria	Ethiopia	Biruksew et al. (2018)
Zygophyllaceae (1)	*Balanites rotundifolia*	Leaves	Malaria	Ethiopia	Asrade et al. (2017)

a Number of species studied by family in parentheses.

b Antimalarial activity evaluated against gametocytes.

In this review natural products mostly from medicinal plants were considered active when inhibition of parasite growth was equal to or above 30% (the lower limit for moderately active products); those below 30% were considered to be inactive (Carvalho et al., 1991; Andrade-Neto et al., 2003; Coutinho et al., 2013; Induli et al., 2013; Musila et al., 2013; Nguta and Mbaria, 2013; Bantie et al., 2014; Upadhyay et al., 2014; Aguiar et al., 2015; Rocha e Silva et al., 2015; Satish et al., 2017).

## Results and Discussion

The scientific names of the plant species tested, their botanic families, parts of the plants fractionated, the ethnomedicinal uses and the countries where the studies were performed are shown in Table 2. Among the 72 articles, 54 of them evaluated the antimalarial activities of one plant species each while 18 of them evaluated two or three plant species each, giving rise to a total of 90 plant species, some of which were studied by more than one author. The three most studied families were Fabaceae, Apiaceae and Lamiaceae. The plant species evaluated for antimalarial activities corresponded to 44 botanical families; the parts of the plants tested were aerial parts, leaves, leaf latex, rhizomes, roots, root bark, stem bark, whole stem, branches, twigs, petiole, cortex, flower, fruits, fruit pulp, unripe fruits, and whole plant (Table 2).

**FIGURE 6:**
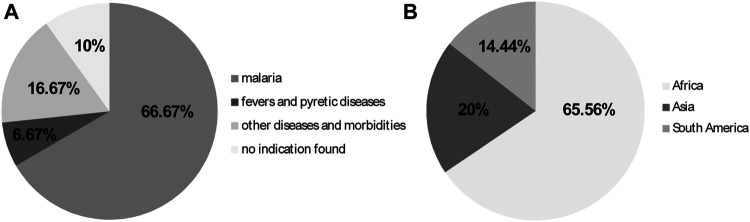
**(A)** Ethnopharmacological uses of plant species evaluated against *Plasmodium* species in mice from 2011 to 2020; **(B)** Continents where tests were performed, presumably, where the plants occur or are natives.

A total of 60 of the 90 medicinal plant species (66.67%) tested in mice with rodent *Plasmodium* species were recommended for the treatment of malaria and/or malaria prevention, as well as for other ailments; six species (6.67%) were used against fever in general, which happens to be the main acute symptom of human malaria. A total of 15 species (16.67%) had an ethnopharmacological recommendation for other diseases and morbidities rather than malaria, while nine species (10%) did not have their ethnobotanical uses mentioned (Figure 6A).

Further information about the plant species studied are depicted in Table 2. Most studies regarding the antimalarial activities of plant species using animal models, were performed presumably where the plant species occur and/or are native: 59 of the species were in the African continent (65.56%), 18 species were in Asia (20%) and 13 species were in South America (14.44%) (Figure 6B).

**FIGURE 7:**
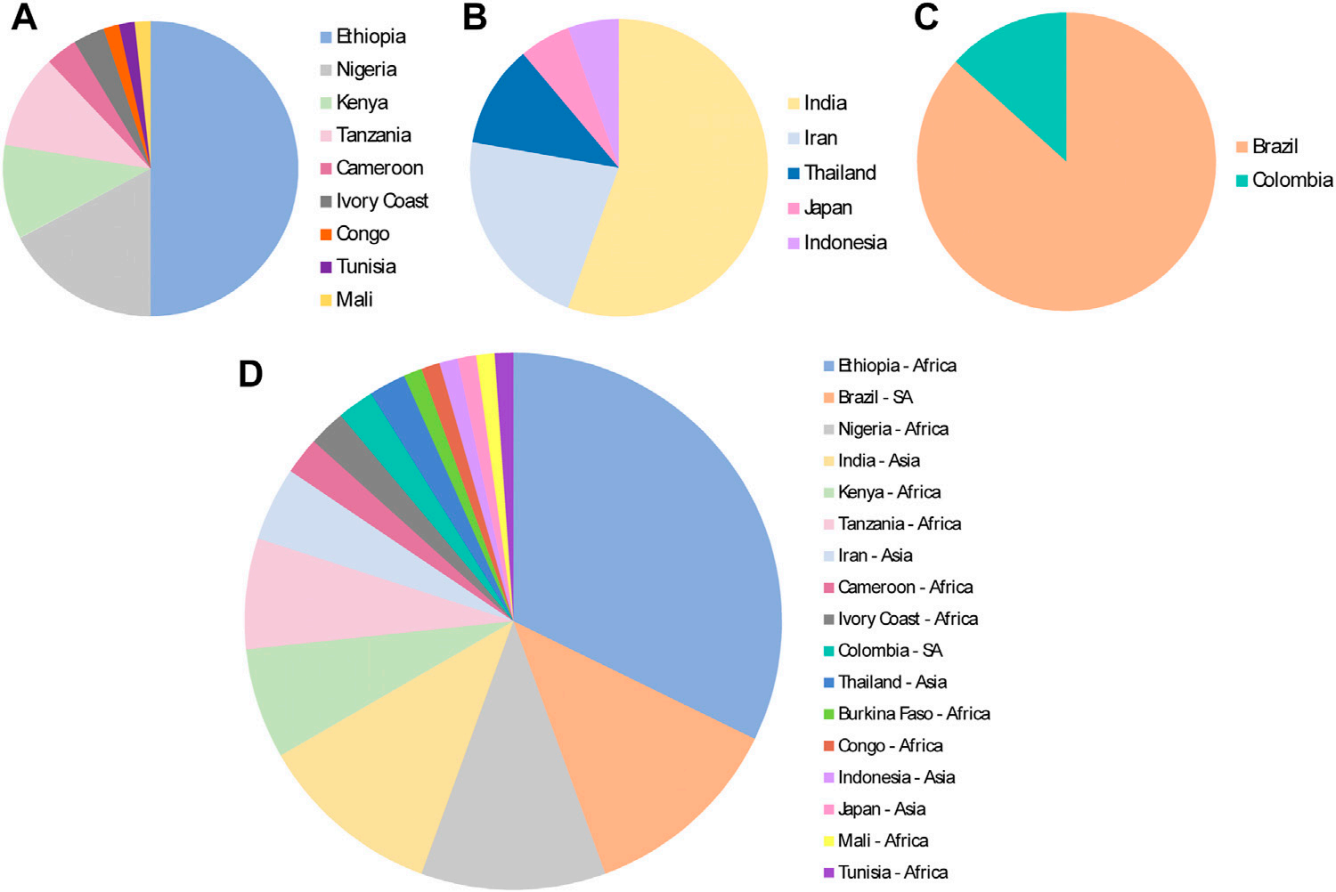
Continents and countries where medicinal plants were evaluated for antimalarial activities in the murine malaria model, from 2011 to 2020, based on total number of plant species evaluated. **(A)** Africa; **(B)** Asia; **(C)** South America (SA); **(D)** All different countries and regions.

Regarding the countries where the studies were performed in mice infected with malaria parasites, an African country had the highest number, that is Ethiopia (Africa, *n* = 29), followed by Brazil (South America, *n* = 11), India (Asia, *n* = 10) and Nigeria (Africa, *n* = 10) (Figure 7).

Other details of the *in vivo* tests described in the original articles are summarized in Table 3. As expected, most medicinal plants exhibited antimalarial activities against *Plasmodium* species in mice experimentally infected (*P. berghei* or *P. yoelli*), though some active doses were too high. These doses are not feasible under clinical settings, making such extracts of no clinical relevance. The results validated the ethnobotanical uses of several plants in malaria endemic countries as they reduced parasitaemia and were well tolerated by the uninfected mice (Table 3).

**TABLE 3:** Antimalarial activities and toxicities of medicinal plants evaluated in animal models from 2011 to 2020.

Plant species	Parasitemia Inhibition with each extract and dose used for the treatment of the malaria infected mice (dose in mg/kg body weight)	Increased survival of the malaria infected mice	Safe dose to non-infected mice (mg/kg body weight)
*Acacia karroo*	Methanolic leaf extract 57% (500)	NE	NE
*Acacia nilotica*	Aqueous root fraction F-1 77% (100)	Yes	NE
*Acanthus polystachyus*	Methanolic roots extract 33% (200), 51% (400) (Derebe and Wubetu, 2019); methanolic leaves extract 34% (200), 49% (400) (Kifle and Atnafie, 2020)	Yes	2,000
*Acokanthera schimperi*	Methanolic leaf extract 37% (600)	Not improved	2,000
*Adansonia digitata*	Aqueous stem bark extract 60% (100), organic stem bark extract 33% (100)	NE	LD_50_ >1,000 µg/ml
*Aloe* sp	Ethanolic leaf extract 74% (650), aqueous leaf extract 58% (650)	Not improved	3,000
*Aloe pirottae*	Aqueous latex extract 39% (400), 47% (600)	Yes	NE
*Aloe weloensis*	Aqueous leaf latex extract 42% (200), 67% (400)	Yes	2,000
*Ajuga bracteosa*	Ethanolic leaf extract 67% (250), 80% (500), 85% (750)	Yes	NE
*Ajuga integrifólia*	Methanolic aerial part extract 35% (800)	Yes	2,000
*Andropogon leucostachyus*	Aqueous aerial part extract 71% (250)	Not improved	NE
*Anthocleista djalonensis*	Hydroethanolic stem bark extract 36% (200), 48% (400), 71% (600)	Yes	NE
*Aspidosperma nitidum*	Ethanolic wood bark extract 48% (250); chloroform fraction 43% (250); methanolic extract >67% (100); fraction from methanolic extract >48% (100); FO III 66% (50); FO IV 65% (50); precipitate fraction 57% (50)	Yes	NE
*Aspidosperma olivaceum*	Acidic stem bark fraction 79% (100); 58% (200)	Not improved	NE
*Aspidosperma pyrifolium*	Root bark extract 79% (100), root extract 75% (100); aqueous stem bark fraction 93% (100); alkaloid-rich stem fraction 79% (100)	NE	NE
*Aspidosperma ramiflorum*	Methanolic stem bark neutral precipitate extract 66% (250) and 53% (500)	Yes	NE
*Azadirachta indica*	Ethanolic leaf extract 55% (650)	Not improved	3,000
*Balanites rotundifolia*	Methanolic leaf extract 37% (100), 42% (200), 67% (400)	Yes	2,000
*Bombax buonopozense*	Aqueous root bark extract 81% (50), 86% (100), 93% (200)	Yes	5,000
*Caesalpinia bonducella*	Dichloromethane root extract 38% (400)	Yes	NE
*Caesalpinia pluviosa*	Ethanolic stem bark Fraction F4 79% (50)	NE	NE
*Calotropis gigantea*	Methanolic leaf extract 41% (100); 52% (200); 65% (400); 72% (800); 74% (1,000)	Yes	NE
*Canthium glaucum*	Aqueous root extract 32% (100), organic extract 44% (100)	NE	LD_50_ >1,000 µg/ml
*Chenopodium ambrosioides*	Hydroalcoholic leaf extract 53% (5)	Yes	NE
*Citrullus colocynthis*	Methanolic fruit extract 65% (50)	Yes	NE
*Commiphora africana*	Dichloromethane bark extract 64% (400)	Yes	NE
*Copaifera reticulata*	Oleoresin 96% (100), 93% (200)	Yes	2,000
*Coptis japônica*	Aqueous rhizome extract 50% (122); coptisine chloride 89% (365)	NE	NE
*Cordia africana*	Methanol leaf extract 51% (600), butanol fraction 56% (400), chloroform fraction 45% (400)	Yes	2,000
*Croton macrostachyus*	Methanol leaf extract 44% (200), 78% (400), 91% (600); chloroform fraction 49% (200), 66% (400), 76% (600); methanol fraction 37% (200), 53% (400), 64% (600); aqueous fraction 39% (600) (Bantie et al., 2014); methanolic leaf extract 34% (600), aqueous leaves extract 31% (400), 51% (600) (Mohammed et al., 2014)	Yes	5,000 or 2,000
*Cucumis metuliferus*	Chloroform extract 46% (300), 80% (600), 99% (1,500), methanolic extract 37% (300), 59% (600), 89% (1,500), ethyl acetate extract 31% (300), 64% (600), 84% (1,500)	Yes	NE
*Dichrostachys cinérea*	Dichloromethane bark extract 53% (400)	Yes	NE
*Dicoma tomentosa*	Ethanolic 50% whole plant extract 90% (300); aqueous whole plant extract ∼80% (300); methanol whole plant extract ∼40% (100); methanolic + ethanolic 50% extract ∼40% (100)	NE	NE
*Dodonaea angustifólia*	n-butanol root fraction 38% (200), 56% (400), 68% (600); chloroform root fraction 37% (400), 42% (600)	Yes	2,000
*Daucus virgatus* ^a^	Methanolic aerial part Daucovirgolide G 92% (50 µg/ml)	NE	NE
*Echinops hoehnelii*	Methanolic root extract 69% (200), 79% (400); dichloromethane fraction 34% (200), 43% (400); fractons from 5-(penta-1, 3-diynyl)-2-(3,4-dihydroxybut-1-ynyl)-thiophene 43.2% (50) and 50% (100); 5-(penta-1,3-diynyl)-2-(3-chloro-4-acetoxy-but-1-yn)-thiophene 33% (100)	Yes	2,000
*Echinops kebericho*	Ethanolic rhizome extract 58% (500) (Toma et al., 2015); methanolic root extract 35% (500), 50% (1,000) (Biruksew et al., 2018)	Yes	5,000
*Entandrophragma cylindricum*	Ethyl acetate stem bark extract 99% (250), 100% (500)	Yes	NE
*Euterpe oleracea*	Aqueous fruit pulp fraction 1 (total phenolics) 89% (20)	Yes	NE
*Fagara zanthoxyloides*	Methanolic leaf extract 82% (200), 91% (400), 96% (600)	NE	5,000
*Ferulago angulata*	Ethanolic aerial part extract 30% (100), 40% (300), 50% (600); superosin peroxidase 40% (10), 50% (30), 50% (75), 75% (100)	Yes	NE
*Ficus thonningii*	Hexane leaf extracts 65% (100), 71% (200), 76% (300), 83% (400), 85% (500)	Yes	NE
*Gardenia ternifolia*	Methanolic root bark extract 33% (200), 47% (400), 59% (600); butanol fraction 31% (200), 42% (400), 51% (600); chloroform fraction 31% (400), 41% (600)	Yes	2,000
*Glycyrrhiza glabra*	Butanolic root extract, 18β-glycyrrhetinic acid 68–100% (62.5–250)	NE	NE
*Grewia trichocarpa*	Aqueous root extract 36% (100)	Not improved	LC_50_ of 545.8 µg/ml
*Gynostemma pentaphyllum*	Aqueous leaf extract 45% (500), 50% (1,000), 55% (2,000)	NE	4,000
*Heinsia crinita*	Dichloromethane stem bark extract 49% (300)	NE	NE
*Helianthus annuus*	Ethanolic extracts: root 36% (1), 48% (10), 64% (100), 72% (250), 79% (400), 63% (800); stems 40% (100); seeds 42% (100); flowers 36% (100) (Ekasari et al., 2019); leaf 49% (1), 76% (10), 82% (100) (Ekasari et al., 2020)	Yes	NE
*Holarrhena pubescens*	Methanolic root extract 32% (400), 43% (800)	Not improved	NE
*Hypoestes forskalei*	Methanolic leaf extract 47% (200), 51% (400), 56% (600)	Yes	2,000
*Icacina senegalensis*	Ethanolic root extract 81% (50), 86% (100), 92% (200)	Yes	NE
*Indigofera spicata*	Methanolic root extract 17% (200), 35% (400), 53% (600)	Yes	NE
*Kniphofia foliosa*	Rhizome extract, Knipholone anthrone 30% (100)	NE	NE
*Leonotis ocymifolia*	Hydroalcoholic extracts of leaf 7% (100), 23% (200), 37% (400), 41% (800)	Not improved	2,000
*Lippia kituiensis*	Methanolic leaf extract 36% (300), 69% (600), 75% (1,500); Chloroform leaf extract 46% (300), 70% (600), 94% (1,500); Ethyl acetate leaf extract 42% (300), 70% (6,000), 95% (1,500)	Yes	NE
*Lophira alata*	Hexane leaf extract 37% (200), 60% (300), 69% (400), 74% (500)	Yes	NE
*Markhamia tomentosa*	Aqueous leaf extract 46% (250), 43% (500), 73% (800)	Yes	NE
*Melia azedarach*	Methanolic aerial part extract 32% (800)	Yes	2,000
*Moringa oleifera*	Aqueous leaf extract 35% (50), 40% (100), 50% (200)	NE	4,000
*Murraya koenigii*	Aqueous leaf extract 62% (350), 72% (750), 77% (1,000); ethyl acetate leaf extract 87% (600); myristic acid 83% (100); β-caryophyllene 88% (100)	Yes	NE
*Myrica salicifolia*	Methanolic root extract 38% (100), 51% (200), 59% (400)	Yes	2,000
*Nuxia congesta*	Hydromethanolic leaf extract 41% (500), 45% (750), 58% (1,000); aqueous fraction 45% (600)	Yes	5,000
*Ocimum lamifolium*	Aqueous leaf extract 36% (600)	Yes	2,000
*Ocimum sanctum*	Aqueous leaf extract 44% (750), 36% (1,000)	Yes	NE
*Ocimum suave*	Chloroform-methanolic leaf extract 55% (100)	NE	2,000
*Olea europaea*	Methanolic stem bark extract 30% (200), 43% (400), 52% (600); butanol fraction 35% (200), 45% (400), compound C 38% (200); methanolic leaf extract 50% (200), 55% (400), 58% (600). Fractions: chloroform 32% (200), 36% (400), 38% (600); butanol fraction 41% (200), 46% (400), 51% (600)	Yes	2,000
*Osyris quadripartita*	Chloroform leaf extract 41% (600)	Yes	2,000
*Periploca linearifolia*	Methanolic stem bark extract 38% (200), 43% (400), 57% (600)	Yes	2,000
*Phyllanthus nivosus*	Ethanolic leaf extract 83% (100), 81% (200)	NE	NE
*Physalis alkekengi*	Methanolic fruit and leaf extract 58% (100)	Yes	NE
*Picramnia latifolia*	Ethanolic bark/petiole extract 51% (1,000)	NE	2,000
*Picrolemma huberi*	Ethanolic cortex extract 93% (150)	NE	2,000
*Piper peltatum*	Chloroformic-ethanolic root extract, 4-Nerolidylcatechol 34% (400), 63% (600)	Yes	NE
*Plectranthus barbatus*	Chloroformic-methanolic root bark extract 79% (100)	NE	2,000
*Polyalthia longifólia*	Aqueous leaf extract 53% (800)	NE	NE
*Pongamia pinnata*	Methanolic bark extract 84% (1,000)	Yes	NE
*Psidium guajava*	Aqueous extract: unripe fruits 30% (350), 65% (750), 62% (1,000); leaves 74% (350), 80% (750), 86% (1,000)	Yes	NE
*Rubus ellipticus*	Methanolic seed extract 64% (500)	NE	NE
*Solanum nigrum*	Methanolic fruit extract 61% (100)	Yes	NE
*Sorindeia juglandifolia*	Methanolic fruit extract, compound 1 - 2,3,6-trihydroxy benzoic acid (1) 54% (50), 70% (100)	NE	7,000
Strychnos mitis	Aqueous leaf extract 75% (400), 96% (600); hydro-methanolic leaf extract 36% (200), 81% (400), 94% (600)	Yes	2,000
*Syzygium cumini*	Methanolic leaf extract 52% (500)	NE	NE
*Tamarindus indica*	Aqueous fruits extract 81% (650)	Not improved	3,000
*Terminalia brownii*	Methanol bark extract 33% (100), 47% (200), 60% (400); aqueous bark extract 39% (200), 51% (400)	Yes	2,000
*Terminalia macroptera*	Leaves ethanolic extract 37% (100), roots ethanolic extract 46% (100)	Not improved	2,000
*Trichilia heudelotii*	Aqueous stem bark extract 40% (800)	NE	NE
*Xylopia amazônica*	Aqueous leaf and branch extract 52% (250)	Not improved	NE
*Zanthoxylum chalybeum*	Aqueous leaf extract 78% (100)	NE	2,000
*Zingiber officinale*	Methanolic root extract 33% (1,000)	Yes	NE
*Ziziphus mauritiana*	Hydroethanolic stem bark extract 39% (200), 66% (400), 89% (600)	Yes	NE

NE, not evaluated.

a Antimalarial activity evaluated in gametocytes.

The active doses of the extracts from medicinal plants tested in mice infected with rodent malaria species ranged from 1 to 6,000 mg/kg, although there was no clear relationship between doses and percentage inhibition of parasite growth, in several cases. The mean survival time was assessed for 65 (72.2%) of the species evaluated; for 55 of them (84.6%), there was an increase in the survival time of the treated mice while for 10 plant species (15.4%) there was no change in the time of animal survival, as compared to the untreated malaria controls (Table 3). The acute toxicity was evaluated for 40 of the 90 plant species; the LD_50_ values for 37 species (41.1%) were 2,000 mg/kg body weight or above (Table 3), and six of the plant species had LD_50_ values equal to or higher than 5,000 mg/kg body weight, suggesting that these plant extracts were not toxic (Lorke, 1983).

The most active species was *Heliantus annuus* (popularly know as sunflower) in tests using ethanolic extract of the leaves, which caused a high suppression of parasitemia (49, 76, and 82% using oral doses of 1, 10 and 100 mg/kg, respectively). Its mechanism of action was through the *in vitro* inhibition of heme polarization by the parasites in a dose dependent manner. Extracts of other parts of the plant species, including roots, stems and flowers (Table 3) were also highly active (Ekasari et al., 2019; Ekasari et al., 2020), but the toxicity of the extracts was not evaluated, perhaps because the sunflower oil from seeds is largely used all over the world in food preparation.

Medicinal plants are often used in health care delivery as complementary and/or alternative medicines against human malaria, specially in poorer areas of the African countries. Such use may be important, especially in conjunction with other health measures like mosquito bed-nets, repellents, and/or treatments with an antimalarial, including the inexpensive chloroquine pills, that costs about 10 US cents per course of treatment for an adult (Institute of Medicine, 2004).

The usefulness of traditional medicinal plants as antimalarials, as judged from the work herein analysed, is relevant, though some of the experimental work have not confirmed a dose response activity in the rodent malaria model, suggesting that they lack relevant activity. In addition and also importantly, in most studies organic solvents were used for the extraction of the plant materials tested, despite the fact that it has been recommended that only water and ethanol should be used in traditional preparations (Willcox et al., 2011). Therefore, with the present analysed data, it is difficult to evaluate the real pharmacological usefulness of medicinal plant extracted in organic solvents to serve as future bioproduct for the development of antimalarials.

### Tests of Medicinal Plants From Brazil Against Experimental Rodent Malaria

**FIGURE 8:**
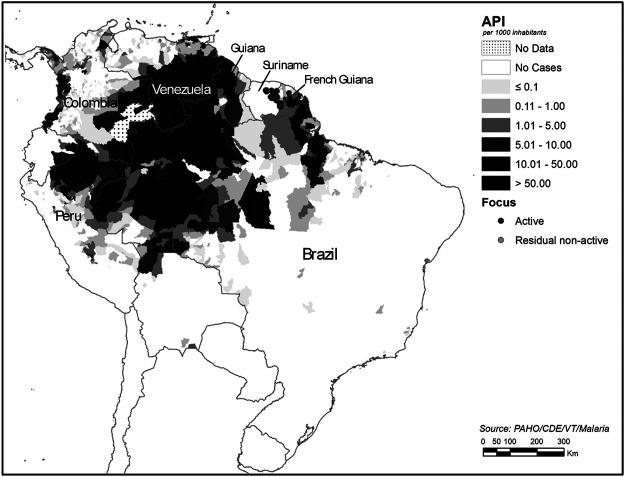
Malaria by Annual Parasite Index (AP1) in the Americas (Pan American Health Organization, 2017), adapted by the authors. Source: https://www.paho.org/hq/index.php?option=com_topics&view=article&id=33&Itemid=40757&lang=en.

In Brazil, plants and other natural products are rarely used to treat or prevent malaria. This, in part, is due to the fact that the Public Health system provides diagnosis and treatment of the disease free of charge. The specific diagnosis is promptly provided by the public health system of the Ministry of Health in Brazil, followed by treatment with antimalarials. In addition, human malaria is of compulsory notification to the Public Health system, that also controls the antimalarial drug distribution, under the State governments in Brazil (Ministério, 2020). Treatment is only provided after the parasite species has been parasitologically confirmed, and it varies according to the diagnosis of the species of parasite, which at present is mainly *P. vivax* followed by *P. falciparum*. The malaria endemic areas are restricted to the Amazon region in Brazil and its neighbouring countries in South America: Colombia, Peru and Venezuela and the Guyanas (Figure 8).

In the Amazon region, the Quilombolas are endowed with extensive experience in the use of medicinal plants, as they have centuries of close contact with and dependence on local biodiversity as a means of livelihood and therapeutic resources. This fact makes the traditional communities attractive for groups conducting ethno-directed studies for medicinal plants used against malaria and other diseases (Oliveira et al., 2015). The popular uses of one species of medicinal plant, *Ampelozyzyphus amazonicus,* known as “Indian beer,” has been previously described, and found to be frequent among the indigenous people in the State of Amazon (Botelho et al., 1981; Brandão et al., 1985; Carvalho et al., 1991; Brandão et al., 1992; Krettli et al., 2001; Oliveira et al., 2011), and also common among the Quilombolas (Oliveira et al., 2011, 2015).

It has been demonstrated that the ethanolic extracts of *A. amazonicus* target the sporozoites, the infective form inoculated through the mosquito bite; *in vitro* and *in vivo* models were used in the study (Andrade-Neto et al., 2008). The animals treated with ethanolic extracts by oral route, prior to sporozoite inoculation intravenously, had a significantly prolonged malaria pre-patent period (which is the time relapsed between sporozoite inoculation and detection of parasitemia in the experimental animals). The treated mice had a lower parasitemia and a prolonged survival time, as compared to the untreated control mice infected with sporozoites. Additional *in vitro* tests clearly confirmed the *in vivo* results: sporozoites pre-incubated with the plant extracts, were less infective to host cells, as compared to control sporozoites in culture medium. In conclusion, the “Indian beer” ethanolic extract was shown to be a potent prophylactic against malaria (Andrade-Neto et al., 2008). In other studies, it was clearly shown that the extracts of “Indian beer” were inactive against the blood forms of the parasite in mice (Botelho et al., 1981; Brandão et al., 1992).

Unfortunately, very few groups studying the experimental activity of medicinal plants in the world have facilities to produce sporozoites for the *in vivo* and *in vitro* tests. Thus, compounds active against these forms, or against the intrahepatic parasites developed from sporozoites will not be discovered until such tests are more easily performed with medicinal plants aiming at new antimalarials. Our group has developed an experimental model to test extracts from plants against the sporogonic stages in mosquitoes as well as the tissue cycle of sporozoite development, using an avian malaria parasite *P. gallinaceum* and mosquitoes *Aedes*, which are susceptible to the species (Carvalho et al., 1992).

Another medicinal plant species frequently used to treat fevers and malaria in Brazil is *Bidens pilosa*, which is active against malaria and was listed as an antimalarial medicinal plant of interest to the Unified Health System in Brazil, largely based on experimental studies of our group (Andrade-Neto et al., 2004; Oliveira et al., 2004; Palhares et al., 2015). The ethanolic crude extracts from *B. pilosa* caused 60% reduction in parasitaemia at doses of 250 mg/kg in mice infected with *P. berghei*; importantly, the ethanolic extracts from *B. pilosa* were similarly active *in vitro* against *P. falciparum* chloroquine-resistant (clone W2) and chloroquine-sensitive parasites (clone D6) (Andrade-Neto et al., 2004).

Essential oils (EOs) have been considered as an important class of antimalarial natural products with low molecular weight components, rich in monoterpenes and sesquiterpenes, found specially in plants native to Northeast Brazil (Mota et al., 2012). Thus, EOs obtained from *Vanillosmopsis arborea* (Asteraceae) were active sub-cutaneously, causing up to 47% inhibition of parasitemia in mice. EOs present in *Lippia sidoides* Cham. (Verbenaceae) and in *Croton zehntneri* (Euphorbiaceae) were active by oral route, causing 43–55% malaria growth inhibition, respectively, although no dose response activity was observed. The active EOs had monoterpene and phenylpropanoid compounds like estragole, α-bisabolol and thymol active *in vitro* against *P. falciparum* but not tested in mice with malaria (Mota et al., 2012). Another plant that contains an oleoresin rich in sesquiterpenes and diterpenes, but with high *in vivo* activity, is *Copaifera reticulata*, a tree distributed throughout the Amazon region, which has β-caryophyllene as its major compound, and caused a reduction of 93% in parasitemia when it was tested in mice (de Souza et al., 2017).

A high antimalarial activity has been described in the extracts of another 11 Brazilian medicinal plants, namely, *Chenopodium ambrosioides* (Cysne et al., 2016), *Xylopia amazonica* (Lima et al., 2015), *Aspidosperma nitidum* (Coutinho et al., 2013), *A. olivaceum* (Chierrito et al., 2014), *A. pyrifolium* (Ceravolo et al., 2018), *A. ramiflorum* (Aguiar et al., 2015), *Euterpe oleracea* (Ferreira et al., 2019), *Caesalpinia pluviosa* (Kayano et al., 2011), *Copaifera reticulata* (de Souza et al., 2017), *Piper peltatum* (Rocha e Silva et al., 2011) *and Andropogon leucostachyus* (Lima et al., 2015); all of them have been described as active against experimental malaria in mice and also *in vitro* against *P. falciparum*. The cited genus *Aspidosperma* spp, was the most studied among the Brazilian plants in the last 10 years, and has been considered for further studies in drug development; the species *A. pyrifolium* (at 100 mg/kg) and *A. olivaceum* (at 100 and 200 mg/kg) were the most potent *in vivo* against *P. berghei* murine malaria, with a parasitemia reduction of 75–93% for *A. pyrifolium* (Ceravolo et al., 2018), and 58 and 79% for *A. olivaceum* respectively (Chierrito et al., 2014) (Tables 1, 2).

### Natural Products Isolated From Medicinal Plants and Their Activities

Of all the antimalarial medicinal plants studied from 2011 to 2020, very few have their active principles isolated and characterized, such as *Coptis japonica*, *Heinsia crinata*, *Piper peltatum* and *Murraya koenigii*. Some of the active principles have been previously isolated from other plants while others are novel. Most of the studies carried out on the active principles are mainly *in vitro* studies. This is partly due to the fact that these compounds were isolated in small amounts that allowed only *in vitro* studies, not a main focus of this review. To overcome such bottle-neck, it is important to start the process of isolation of compounds using large amount of plant materials. The isolated compounds evaluated for *in vivo* antimalarial activities were: 1) Coptisine Chloride from *Coptis japonica*, (Teklemichael et al., 2020); 2) 4-Nerolidylcatechol isolated from *Piper peltatum* (Rocha e Silva et al., 2011); and 3) Myristic Acid and β-Caryophyllene from *Murraya koenigii* (Kamaraj et al., 2014) (Tables 2, 3).

In the studies with *Piper peltatum*, very high doses of 4-Nerolidylcatechol, the active principle (400 and 600 mg/kg body weight), have been tested, resulting in chemosuppression of 34.4 and 63.1%, respectively, in *P. berghei* NK65-infected mice (Rocha e Silva et al., 2011). Such high doses are not clinically feasible for human use; thus, efforts should be directed towards the development of compounds with higher activities at low doses as antimalarial drugs.

The 4-day suppressive test (Peters, 1965) was used for all the *in vivo* studies. However, one of the studies failed to determine the parasitaemia of day 4 post-inoculation, which is very important for identifying the fast acting compounds against malaria. It is only the slow acting compounds that are left till days 5, 6, and 7 post-inoculation (Fidock et al., 2004).

One of the compounds, Coptisine Chloride, was administered intraperitoneally, which is not the conventional route of administration for *in vivo* studies in murine malaria. The pharmacokinetic parameters of the drugs administered via such route are different from those of conventionally used routes of administration and therefore, they are not applicable to humans (Teklemichael et al., 2020).

Some pure compounds such as Aspidoscarpine, Uleine, Apparicine, and N-Methyl-Tetrahydrolivacine were isolated from *A. olivaceum* (Chierrito et al., 2014), Isositrikine, 10-Methoxygeissoschizol and Ramiflorine were isolated from *A. ramiflorum* (Aguiar et al., 2015), and the bisindole alkaloid Leucoridine B was isolated from *A. pyrifolium* (Ceravolo et al., 2018); but none of these pure compounds was tested in the experimental mouse model. Braznitidumine was isolated from *A. nitidum* (Coutinho et al., 2013), but it was not active against *P. berghei* in experimentally infected mice.

Only one compound was tested against the life cycle in the mosquito, namely, Daucovirgolide G extracted from the plant *Daucus virgatus*; it is impressive that it inhibits 92% of the early sporogonic stages of the parasite at 50 µg/ml (Sirignano et al., 2019; Table 4). This transmission blocking activity appears to be related to the presence of an intact endocyclic double bond system of the compound, that interacts with the biological target (Sirignano et al., 2019). This structural part is obviously lacking in Daucovirgolide J, which is the reason for its lack of activity (Sirignano et al., 2019).

**TABLE 4:** Acute toxicities of antimalarial compounds isolated from medicinal plants.

Plant species	Names of compounds	Animal	Route	LD_50_ (mg/kg body weight)	References
*Coptis japonica*	Palmatine	Mouse	Oral	1,533.68	Yi et al. (2013)
	Berberine	Mouse	Intraperitoneal	57.6103	Kheir et al. (2010)
			Oral	713.57	Yi et al. (2013)
	Coptisine	Mouse	Oral	852.12	Yi et al. (2013)
			Intravenous	9.0386	Kheir et al. (2010)
*Murraya koenigii*	Myristic Acid	Mouse	Intravenous	43	Oro and Wretlind (1961)
*Piper peltatum*	4-Nerolidylcatechol	Mouse	Oral	673.22	Mendanha da Cunha et al. (2013)

Africa has a rich flora, ranging from the Savannah to the rainforest, with diversity of phytochemicals and other biomolecules which possess various pharmacological activities, some of which have been extensively reviewed elsewhere (Moyo et al., 2015; Van Wyk, 2015; Ahmed et al., 2018; Oguntibeju, 2018; Van Vuuren and Frank, 2020). Many plants have been reported to be used for the treatment of malaria in the African continent (Soh and Benoit-Vical, 2007; Pillay et al., 2008; Adebayo and Krettli, 2011; Maranz, 2012; Chinsembu, 2015; Memvanga et al., 2015; Omara et al., 2020). As the use of *Cinchona* species (from which quinine was isolated) for the treatment of malaria dates back to the 1700s (Guerra, 1977; Lee, 2002), the use of some plants, such as *Azadiractha indica* and *Alstonia broonei*, for the treatment of malaria, indigenously, in the African continent dates back to centuries, though this has not been properly documented. More recently, the efficacies of some of these medicinal plants have been scientifically authenticated and their active principles isolated, with the mechanisms of action of some of them delineated. However, none of the isolated compounds has been developed into drugs to be used clinically for the treatment of malaria, as reviewed below.

The mechanisms of action of most isolated compounds from the medicinal plants are unclear and/or were only studied *in vitro* against *P. falciparum*. This is the case of Coptisine, isolated from *Coptis japonica*, which exhibits its activity by inhibiting *P. falciparum* dihydroorotate dehydrogenase, an enzyme required for the synthesis of pyrimidine in the parasite (Lang et al., 2018). The authors reported that the kinetic parameters obtained revealed that coptisine was an uncompetitive inhibitor of the enzyme. Berberine, isolated from *Coptis japonica*, has been shown to inhibit telomerase activity in *P. falciparum* (Sriwilaijareon et al., 2002), but there are no documents of studies with their activities in the malaria infected mice.

The chemical structure and molecular formula of each isolated compound are shown in Table 5.

**TABLE 5:** Some antimalarial compounds isolated from medicinal plants.

Isolated compound	Molecular formula	Chemical structure
Coptisine chloride	C_19_H_14_ClNO_4_	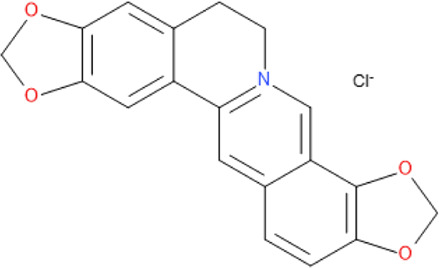
4‐Nerolidylcatechol	C_21_H_30_O_2_	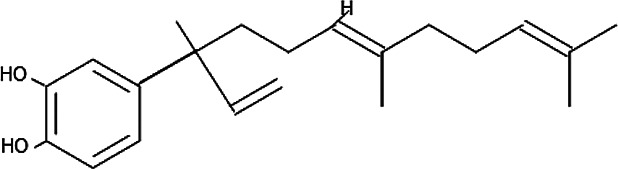
Myristic acid	C_14_H_28_O_2_	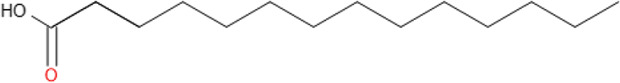
β-Caryophyllene	C_15_H_24_	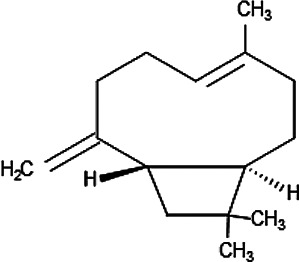
Aspidoscarpine	C_11_H_18_N	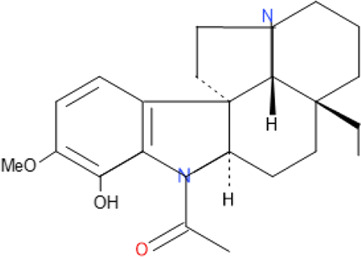
Uleine	C_18_H_22_N_2_	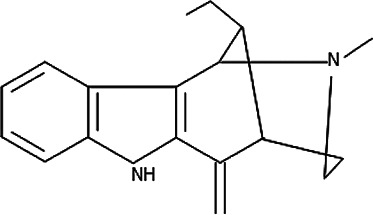
Apparicine	C_18_H_20_N_2_	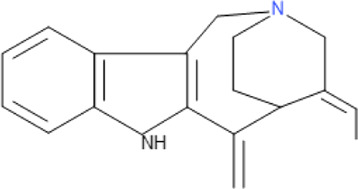
N-Methyl-tetrahydrolivacine	C_18_H_20_N_2_	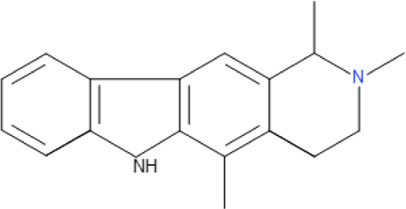
Isositrikine	C_21_H_26_N_2_O_3_	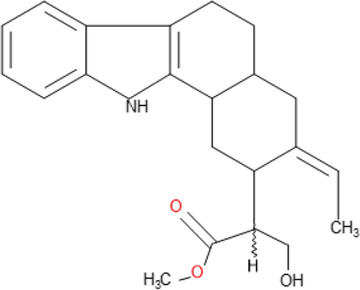
10-Methoxygeissoschizol	C_20_H_26_N_2_O_2_	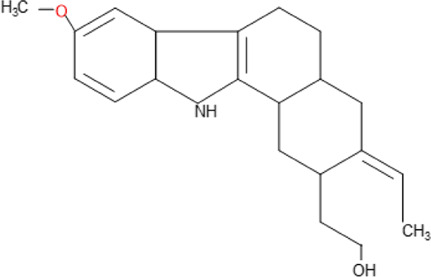
Ramiflorine A and B	C_30_H_34_N_4_O	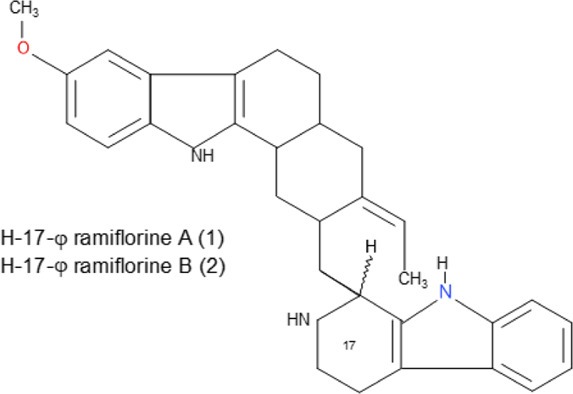
Leucoridine B	C_38_H_42_N_4_	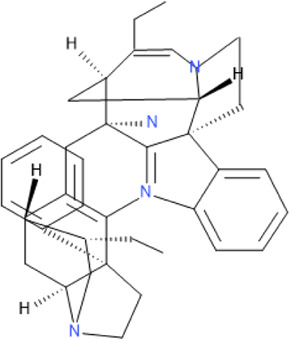
Braznitidumine	C_24_H_32_N_4_O_6_	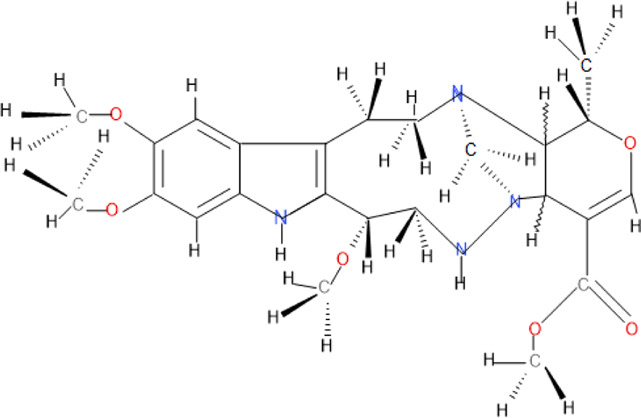
Daucovirgolide G	C_25_H_34_O_7_	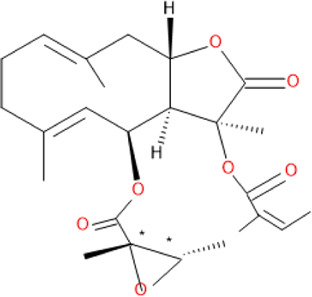
Berberine	C_20_H_18_NO_4_	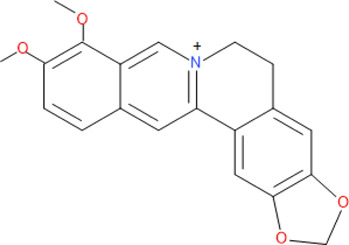

### Acute and Sub-Chronic Toxicity of Some Isolated Compounds

Some of the compounds isolated from *Coptis japonica* (Teklemichael et al., 2020), *Piper peltatum* (Rocha e Silva et al., 2011) and *Murraya koenigii* (Kamaraj et al., 2014) plants have been evaluated for their acute toxicities (Table 4). None of them could be declared safe, having oral LD_50_ values that are less than 5,000 mg/kg body weight (Lorke, 1983). Among the compounds evaluated for acute toxicity, Palmatine was the least toxic with the highest oral LD_50_ value. However, many of the isolated compounds have not been evaluated for their acute toxicity. This may also be due to fact that these compounds were isolated in small amounts which were not sufficient enough to allow their being tested for acute toxicity. Other compounds were tested by intraperitoneal route (Berberine) or by intravenous route (Myristic Acid) which are not routes used traditionally for the treatment of malaria in humans.

The subchronic systemic toxicity, defined as adverse effects occurring after the repeated or continuous administration of a test sample for up to 90 days, does not seem to be related to the extracts that have been used against malaria; furthermore, only Coptisine Chloride was tested *in vivo* (Teklemichael et al., 2020) (Table 3). Other studies have shown that administration of Palmatine, Coptisine and Berberine, at the dose of 156 mg/kg body weight, did not exert nephrotoxic or hepatotoxic effects, though Palmatine significantly increased (*p* < 0.05) plasma total bilirubin concentration compared to control (Yi et al., 2013). However, the authors did not proceed further to identify whether hemolysis, impaired conjugation of bilirubin in the liver or excretion of conjugated bilirubin into the bile duct was responsible for such increase. Many of the compounds have not been evaluated for sub-chronic toxicity or for chronic toxicity. This has limited the determination of the no adverse effect levels (NOAEL) and low adverse effect levels (LOAEL) of the compounds.

### Genotoxicity

Berberine, a compound isolated from *Coptis japonica* (Table 4), has been reported not to be genotoxic for prokaryotic cells (Pasqual et al., 1993); however, it has been reported to be genotoxic to dividing eukaryotic cells by intercalating with the DNA (Jennings and Ridler, 1983). Palmatine has been reported to exert genotoxicity causing DNA double strand breaks, though to a lesser extent than Berberine (Chen et al., 2013). Both compounds have been reported to inhibit topoisomerase I and II activities, thereby inhibiting DNA relaxation and decatenation during replication (Chen et al., 2013). Inhibition of DNA decatenation results in the stabilization of topo II-DNA complexes (Li et al., 2010), thereby enhancing the induction of DNA double strand breaks. Palmatine has been reported to interact with DNA by binding to the groove of DNA (Mi et al., 2015). Knipholone and knipholone anthrone have been reported not to cause DNA damage on their own but knipholone anthrone caused DNA damage in the presence of copper ions through the generation of hydrogen peroxide (Habtemariam and Dagne, 2009). 4-Nerolidylcatechol has been shown to exhibit low genotoxicity compared to negative control. However, 4-Nerolidylcatechol has been reported to protect against cyclophosphamide-induced DNA damage by scavenging the free radicals generated by cyclophosphamide (Valadares et al., 2007).

### Mutagenicity

It has been reported that berberine, in the absence of microsomal fraction S9, was weakly mutagenic in *Salmonella typhimurium* TA98, a frameshift detecting strain (Nozaka et al., 1990). It did not cause an increase in the frequency of point mutation (Pasqual et al., 1993) under conditions in which the frameshift mutation was increased by a factor of 4 in *Saccharomyces cerevisiae* using this model (Pasqual et al., 1993). None of the studies evaluated the mutagenic activities of the compounds using the murine malaria parasite model which analyses the micronucleated cells (Tometsko et al., 1993).

### Carcinogenicity, Reproductive Toxicity and Allergenicity

Several purified compounds exert carcinogenic effects after accumulation, over time, in cellular systems. The searches made on PubMed and PubChem revealed that the above isolated compounds have not been evaluated for carcinogenic and allergenic effects. Their effects on pregnancy, such as absorption of fetus, and teratogenic effects have not been evaluated. This indicates that all the isolated compounds have not fully gone through the process of drug development. Consequently, not a single one of them has been developed into an antimalarial drug, neither been subjected to clinical trials.

## Conclusion and Future Perspectives

Of the seventy one manuscripts published in the past 10 years that evaluated the antimalarial activities of extracts and isolated compounds from plant species in rodent malaria model, most of them aimed the erythrocytic stages of the parasite, which are responsible for the malaria symptoms. Only one compound was tested against the early sporogonic stages of the parasite. In addition, a few active principles were described. All steps of extractions, from the pre-extraction to final extraction are equally important in the study of medicinal plants, and play a critical role in the outcomes, including the product yield and phytochemical characterization; and these steps seem to interfere in the final product activity (Azwanida, 2015). Most of the experimental studies of plant materials used organic solvents, in spite of the recommendation in the specific literature that only water and ethanol should be used in traditional preparations for medical use (Willcox et al., 2011). Taken together, these facts explain the reduced contribution of Africa, in the past 10 years, to the achievement of the Medicine for Malaria Venture objective, which is the development of a new antimalarial drug every 5 years. The reason for this is not far-fetched but in part, it is due to the gross lack of standard facilities for antimalarial drug development in various countries of the African continent, which is responsible for most of the antimalarial tests herein described, especially in countries like Ethiopia and Nigeria, where most of the results have been published in the last 10 years. It is believed that technological advancement in the African continent and the world at large, will certainly go a long way in tackling this fundamental problem, thereby liberating the continent from the scourge of this disease.
